# Trends in HIV-Related Services Offered by Substance Abuse Treatment Facilities

**DOI:** 10.7759/cureus.57400

**Published:** 2024-04-01

**Authors:** Jordyn E Lee, Kyle Murchison, Lillian Hassanein, Darian Peters, Mario Jacomino, George Luck

**Affiliations:** 1 Medicine, Florida Atlantic University Charles E. Schmidt College of Medicine, Boca Raton, USA; 2 Women's and Children's Health, Florida Atlantic University Charles E. Schmidt College of Medicine, Boca Raton, USA; 3 Integrated Medical Science, Florida Atlantic University Charles E. Schmidt College of Medicine, Boca Raton, USA

**Keywords:** needle sharing, substance abuse treatment, hiv testing, hiv transmission, harm reduction, hiv prevention

## Abstract

Introduction

In the United States, persons who inject drugs (PWID) represent an increasingly vulnerable population, with a high risk of HIV transmission related to needle sharing. This paper aims to investigate the availability of HIV-related services within substance abuse treatment facilities while emphasizing the need for implementing comprehensive harm-reduction strategies in such facilities.

Methods

This study explores the prevalence and trends regarding HIV-related services within substance abuse treatment facilities in the United States including testing, counseling, early intervention, and medication provision. Data from the National Survey of Substance Abuse Treatment Services (N-SSATS) were analyzed in order to assess trends in HIV-related services from 2013 to 2020.

Results

Facility response rates revealed an increase in the availability of HIV testing and specialized programs for individuals with HIV. However, there was a contrasting trend with the decline in early intervention and counseling services, only with a slight increase in 2020. Additionally, government-owned facilities demonstrated superior performance in delivering HIV services compared to private facilities.

Conclusion

This study highlights the dire need for implementing routine opt-out HIV testing within substance abuse treatment facilities in order to identify new cases. Additionally emphasized is the importance of early intervention for this at-risk population. To effectively address these challenges, we suggest considering the adoption of the "Seek, Test, Treat, Retain" model as a potential solution. Increasing access to HIV-related services within substance abuse facilities requires enhanced resource allocation as well as integrated programs. Identifying deficiencies in HIV service integration is crucial to enhancing care and reducing HIV transmission among PWID.

## Introduction

In the United States, it is estimated that over 3.6 million individuals, comprising 1.46% of the population, are engaged in injection drug use [1]. This subgroup, often referred to as persons who inject drugs (PWID) may acquire and transmit infectious diseases, notably HIV. The mode of transmission predominantly occurs by sharing needles, syringes, or other drug injection equipment with other infected individuals.

It has been demonstrated that the HIV virus can survive within a used syringe for 42 days when stored at a temperature of 4°C, 21 days at room temperature, and one week if stored above room temperature (27, 32, and 37°C) [2,3]. Such data illustrates the importance of implementing comprehensive harm-reduction strategies.

In addition to the inherent risks of sharing needles, PWID are faced with multiple factors that amplify their susceptibility to HIV infection. High-risk behaviors associated with drug use, combined with immunosuppression secondary to chronic drug use, as well as social challenges such as poverty and homelessness, increase the risk of acquiring HIV in this population. Furthermore, it is observed that PWID have limited healthcare utilization rates and often miss opportunities for early diagnosis and care [4]. The delay in care further magnifies the public health implications of HIV transmission within this group.

In 2019, the Centers for Disease Control and Prevention (CDC) estimated that PWID account for one in 15 of new HIV diagnoses in the United States, and HIV diagnoses among PWID have increased in all 50 states and the District of Columbia [5]. One possible explanation for this trend is the growing opioid and heroin epidemic that has led to increased injection drug use [5,6]. Therefore, targeted interventions and harm reduction strategies for this population are critical for lowering the number of new HIV infections in the United States. In August of 2022, the White House’s Office of National AIDS Policy (ONAP) released the National HIV/AIDS Strategy (2022-2025), which set targets for a 75% reduction in new HIV infections by 2025 and a 90% reduction by 2030. A vital component of this plan is the integration of HIV testing, intervention, and treatment within substance abuse treatment facilities [7].

A systematic review published in 2017 appraised evidence from studies that sought to integrate care for people living with HIV and substance use problems [8]. This review showed that integration models are categorized by location (HIV facilities, substance use facilities, and other facilities), level of integration from micro (integrated care delivered to individuals) to macro (system level integrations), and degree of integration from least (screening and counseling only) to most (care for HIV, substance use and/or other illnesses at the same facility) based on 51 studies from various parts of the world [8]. Our goal was to further determine the current prevalence and trends of HIV-related services offered by substance use facilities in the United States, including HIV testing, HIV counseling and education, early HIV intervention, and HIV medications. In doing so, we hope to identify potential gaps in the identification and treatment of HIV in substance abuse treatment settings.

## Materials and methods

Results from the Substance Abuse and Mental Health Services Administration’s (SAMHSA) annual National Survey of Substance Abuse Treatment Services (N-SSATS) were used for this descriptive study. N-SSATS data is publicly available online through SAMHSA’s website. This survey collects information regarding the operation of substance abuse treatment facilities (e.g., private or public, state or federal government-owned), client demographics, and the services provided by these facilities [9-18].

Since 2013, the N-SSATS has included questions regarding HIV-related services, including the provision of HIV testing, HIV education and counseling, early intervention for HIV, and specialized programs for clients with HIV/AIDS. In 2019 and 2020, additional questions were included in the N-SSATS regarding the provision of medications for HIV treatment such as antiretroviral drugs (e.g., tenofovir, efavirenz, and emtricitabine). For this study, we analyzed the trend data regarding the provision of the aforementioned HIV-related services within substance abuse facilities at a national level from 2013 to 2020 [18]. Additionally, we examined variations in services provided by private non-profit, private for-profit, state government, and federal government facilities in 2020.

## Results

**Figure 1:**
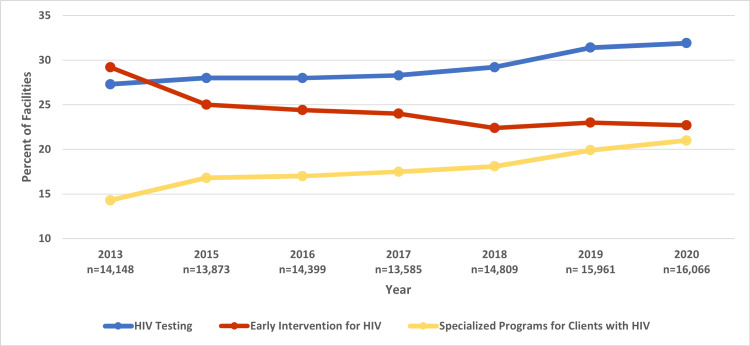
This figure represents the percentage of facilities offering services such as testing, early intervention, and specialized programs for HIV between 2013 and 2020.

Facility survey response rates ranged from 13,857 in 2017 to 14,630 in 2013, with an average of 14,834 facilities responding. Our results demonstrate increases in the percentage of facilities offering HIV testing and specialized programs for clients with HIV/AIDS from 2013 to 2020 (Figure 1). The latter demonstrated the most notable increase, from 2,039 out of 14,148 (14.4%) facilities offering specialized programs for clients with HIV/AIDS in 2013 to 3,374 out of 16,066 (21%) in 2020. A more modest increase in HIV testing was observed, from 4,000 out of 14,148 (27.3%) facilities in 2013 to 5,125 out of 16,066 (31.9%) in 2020. The percentage of facilities offering early intervention for HIV has decreased quite steadily, from 3,185 out of 14,148 (29.2%) facilities in 2013 to 3,647 out of 16,066 (22.7%) in 2020 (Figure 1). Analysis of these trends using chi-square tests revealed statistically significant changes in the provisions of HIV services over the analyzed years. Specifically, the chi-square test for trend indicated a statistically significant increase in the proportion of facilities offering HIV testing over the years $\chi$^2^=99.71, df=6, p,0.001). Further, the chi-square test for trend showed a statistically significant decrease in the provision of early intervention for HIV over the years ($\chi$^2^=130.25, df=6, p<0.001), while a statistically significant increase was observed in the provision of specialized programs for clients with HIV/AIDS over the analyzed years ($\chi$^2^=124.21, df=6, p<0.001).

**Figure 2:**
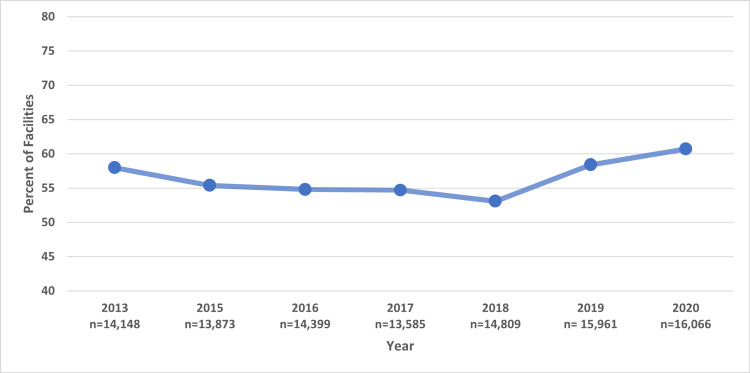
This figure represents the percentage of facilities offering HIV education, counseling, or support between 2013 and 2020.

Over the years analyzed, HIV counseling, education, or support has shown minimal change since 2013 when 8,202 out of 14,148 (58%) facilities reported offering these services (Figure 2). This reached its lowest level in 2018, with 7,862 out of 14,809 (53.1%) facilities providing these services. It has since increased to 9,781 out of 16,066 (60.9%) of facilities offering HIV counseling, education, or support in 2020. Further, a chi-square test was performed to assess the linear trend in the proportion of facilities offering HIV counseling, education, or support over the years. The test revealed a statistically significant linear trend $\chi$^2^ =124.47, df=6, p<0.001), indicating that the observed increase in the proportion of facilities providing such provisions is unlikely to have occurred by chance. 

**Figure 3:**
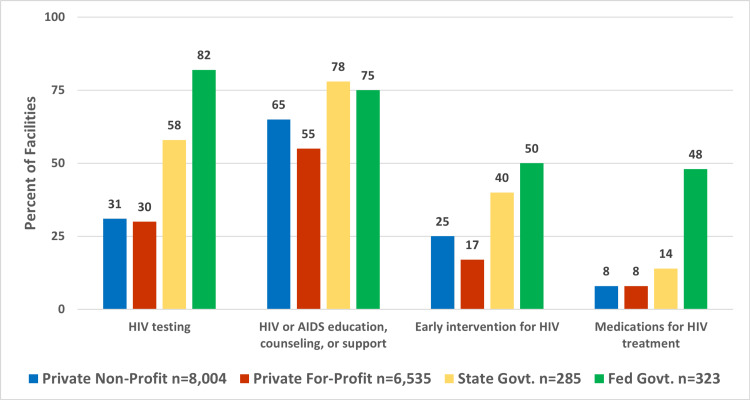
This figure represents the distribution of HIV-related services offered by substance use facilities, categorized by facility type in 2020.

Additionally, it was found that the provision of HIV-related services differed among private non-profit, private for-profit, state government, and federal government facilities (Figure 3). Chi-square tests for independence were conducted to assess the association between the type of facility and the provision of specific HIV services. In general, a greater percentage of government-owned facilities, particularly federal government facilities, offered HIV-related services compared to privately owned facilities. Private for-profit facilities were less likely than non-profit facilities to offer HIV education, support, and counseling, as well as early intervention for HIV. The starkest difference appears in the provision of HIV medications. The chi-square test revealed a significant association between the type of facility and the provision of HIV medications ($\chi$^2^=309.39, df=3, p<0.001). Medications for HIV treatment were provided by 154 out of 323 (48%) federal government facilities, 41 out of 285 (14%) state facilities, and only 1,208 out of 14,539 (8%) privately-owned facilities.

## Discussion

Our results illustrate both reassuring and concerning trends in the provision of HIV-related services by substance abuse treatment facilities in the United States. While HIV testing offered by facilities has increased since 2013, greater than two-thirds of total facilities fail to provide this service. In a 2015 study examining HIV testing in five substance use facilities in Florida, New York, and California, it was found that 30% of clients who opted for free HIV testing had not undergone an HIV test in the past 12 months. This study also found that 5% of these patients tested positive for HIV, highlighting the point that HIV testing at substance abuse treatment facilities has the potential to identify new cases of HIV and reduce further transmission [19]. Routine opt-out testing of clients who present to substance abuse treatment facilities, which provides HIV testing to all clients unless they choose to forgo testing, may increase the number of people who receive testing at facilities that offer this service. A recent study by Bartholomew et al. found that opt-out testing provided at a syringe exchange service program increased HIV/HCV (Hepatitis C virus) testing by 42.4% [20].

Decreases in the percentage of facilities offering early treatments, education, counseling, and support for HIV patients are of particular concern. Early intervention programs for HIV are essential in helping those newly diagnosed with HIV gain access to care and are critical to reducing HIV transmission and disease progression in PWID. The Strategic Timing of Antiretroviral Therapy (START) study found that patients who began antiretroviral therapy (ART) when CD4+ cell counts were above 500 cells per cubic millimeter had a 57% reduced risk of AIDS and serious non-AIDS health outcomes than patients who started ART after CD4+ cell counts dropped below 350 cells/mm3 [21]. Further, education regarding the importance of HIV testing, availability of pre-exposure prophylaxis (PrEP), harm reduction strategies, and treatment of HIV for clients at substance abuse treatment facilities is crucial for the integration of HIV and substance abuse services. 

Furthermore, while the percentage of facilities offering specialized programs for clients with HIV has been increasing, less than 10% of private facilities and 15% of state government facilities offer medications for HIV treatment. The reasons for this are likely multifactorial, including a lack of providers who are willing and/or able to prescribe HIV medications, inadequate funding, and resources to provide these medications, and various barriers to the implementation of ART within substance abuse treatment facilities [22]. Federal government-owned facilities are most likely to provide HIV testing, early intervention for HIV, and HIV medications. The higher provision of HIV services in federal government facilities is most likely related to increased resource allocation and established protocols [23]. However, federal-government-owned facilities are few in number compared to state- and privately-owned facilities.

The "Seek, Test, Treat, Retain" (STTR) model of care for HIV has recently gained traction as a targeted intervention for persons at high risk of HIV infection, including those who use IV drugs. Within this model, providers identify patients who are at high risk for HIV ("seek"), offer HIV testing to these patients ("test"), initiate or facilitate initiation of ART for patients who are positive for HIV ("treat"), and then connect patients to appropriate follow-up for HIV treatment and treatment of comorbid mental or physical health conditions ("retain") [24]. Adoption of this model within substance abuse treatment facilities, particularly privately-owned facilities that appear less likely to offer HIV-related services, would help to curtail HIV transmission among IV drug users. 

This study has several strengths. The N-SSATS surveys all known public and private substance abuse treatment facilities in the United States with a relatively high response rate (89-94% across the surveys included in this study), resulting in a large sample size and data that is representative of substance abuse treatment facilities on a national level [18].

However, there are limitations to this study which are primarily related to the N-SSATS. The N-SSATS survey is voluntary, and as such, not all facilities respond, and data is not adjusted for facility nonresponse. Further, the N-SSATS is a point prevalence survey and represents a "snapshot" of facility responses on a given day, which may change over the course of a year [18].

## Conclusions

Increasing access to HIV testing, early intervention, education, and treatment within substance abuse facilities will require increased resource allocation. Integrated programs that simultaneously address HIV and substance use disorders can optimize the use of scarce resources at the operational level, thereby offering a way to improve access to care, responsiveness to patients’ needs, increase coverage, reduce inequalities, and improve health outcomes. Ultimately, we highlight potential deficiencies in the integration of HIV services in substance abuse facilities to identify where improvements may be made.
